# COVID-19 primary infection, reinfection, and its associated clinical presentations

**DOI:** 10.3389/fpubh.2026.1695310

**Published:** 2026-04-14

**Authors:** Varuni Makam, Vidyavathi B. Chitharagi, Tejashree A., Morubagal Raghavendra Rao, Smitha Chandrashekarappa, Yogeesh D. Maheshwarappa

**Affiliations:** 1 Department of Microbiology, JSS Medical College and Hospital, JSS Academy of Higher Education and Research (JSS AHER), Mysuru, India; 2 Department of Community Medicine, JSS Medical College and Hospital, JSS Academy of Higher Education and Research (JSS AHER), Mysuru, India

**Keywords:** COVID-19, primary infection, reinfection, SARS-CoV-2, vaccination

## Abstract

**Introduction:**

SARS-CoV-2 (Severe Acute Respiratory Syndrome Coronavirus-2), which caused primary COVID-19 infection in millions worldwide, raised significant concerns as viral mutations emerged, leading to reinfections among previously infected and vaccinated individuals. This phenomenon became particularly evident during the second and third waves of the COVID-19 pandemic in India.

**Methods:**

The current study observed the rates of reinfections due to SARS-CoV-2 and their relation to vaccination details by retrospectively analyzing the data of patients who had reinfections during the second and third waves of COVID-19.

**Results:**

Among the 458 study participants, 62/458 (13.54%) with primary infection had confirmed re-infection. Of the 458 people infected with COVID-19 for the first time, 60 (13%) were asymptomatic and 398 (87%) were symptomatic. The most frequently observed symptoms were fever during primary (70.3%) and re-infection (72.58%). A statistically significant association was observed between vomiting symptoms and infection status (*p* < 0.01).

**Conclusions:**

SARS-CoV-2 is re-emerging with new variants and varied presentations, posing a challenge to clinicians regarding specific diagnoses. The symptoms of COVID-19 during primary and re-infection did not differ greatly. Although vaccination protects individuals against reinfections, the exact duration of protection remains unknown and may necessitate booster doses at frequent intervals.

## 1 Introduction

Coronavirus disease (COVID-19) emerged in December 2019 in Wuhan, China, causing over 6 million deaths worldwide and over 500,000 deaths in India. The causative agent, SARS-CoV-2 (Severe Acute Respiratory Syndrome Coronavirus-2), is an RNA virus that predominantly affects the respiratory tract, typically causing mild to moderate disease. However, patients with comorbidities may develop severe forms requiring mechanical ventilation and intensive care, predisposing them to secondary and opportunistic infections (1). Since the pandemic began, numerous cases of reinfection have been documented in both vaccinated and unvaccinated individuals, raising questions about the durability and extent of immunity conferred by vaccination or prior infection. Although our understanding of SARS-CoV-2 infection has advanced considerably, reinfection remains relatively unexplored. Reinfection is defined as a subsequent infection in the same individual occurring after a defined time interval, with evidence of genotypic variance (2).

In clinical practice, reinfection is considered if there is a clinical recurrence of symptoms compatible with COVID-19, accompanied by a positive PCR test (Ct < 35), more than 90 days after the onset of the primary infection, supported by close-contact exposure or outbreak settings, and no evidence of another cause of infection. If there is significant exposure, reinfection should be considered during the first 90 days, if symptoms of the first episode have resolved and two PCR tests were negative before the next episode (2). Infection is considered to have occurred after vaccination if it occurs after immunity develops, which is 2 weeks after vaccination with second dose. This can occur because of a mutation of the strain, which prevents the action of antibodies developed due to vaccination, causing a clinical/subclinical infection (3).

It was believed that individuals who recovered from COVID-19 or those who were vaccinated generated a strong immune response and developed protective immunity. After the first case of documented reinfection with COVID-19 in August 2020, there has been a gradual increase in several cases of reinfection. Although reinfections are caused by different strains, sequencing remains the sole method for identifying the different strains as a cause of reinfection (3). The objectives of this study were to determine the rate of reinfection in COVID-19 patients after a previous infection, to compare the rate of COVID-19 reinfections among vaccinated and non-vaccinated patients, to compare the differences between the clinical presentations of patients with primary infection and reinfection, and to determine the association between the symptoms and the status of infection.

## 2 Methodology

This cross-sectional, hospital-based retrospective study was conducted at a Tertiary care Hospital in Mysuru, South India. The study included patients who tested positive for SARS-CoV-2 during the third wave of the COVID-19 pandemic in January 2022. Data collection was conducted retrospectively from August to September 2022, following approval from the Institutional Ethics Committee of JSS Medical College and Hospital (JSSMC/IEC/050722/27NCT/2022–23).

Written informed consent could not be obtained as the study took place during the COVID-19 pandemic. Therefore, verbal informed consent was obtained from the participants via phone calls, which were recorded on mobile phones. JSS Medical College and Hospital Institutional Ethical Committee approved the verbal informed consent. The study adheres to the Declaration of Helsinki.

**Figure 1:**
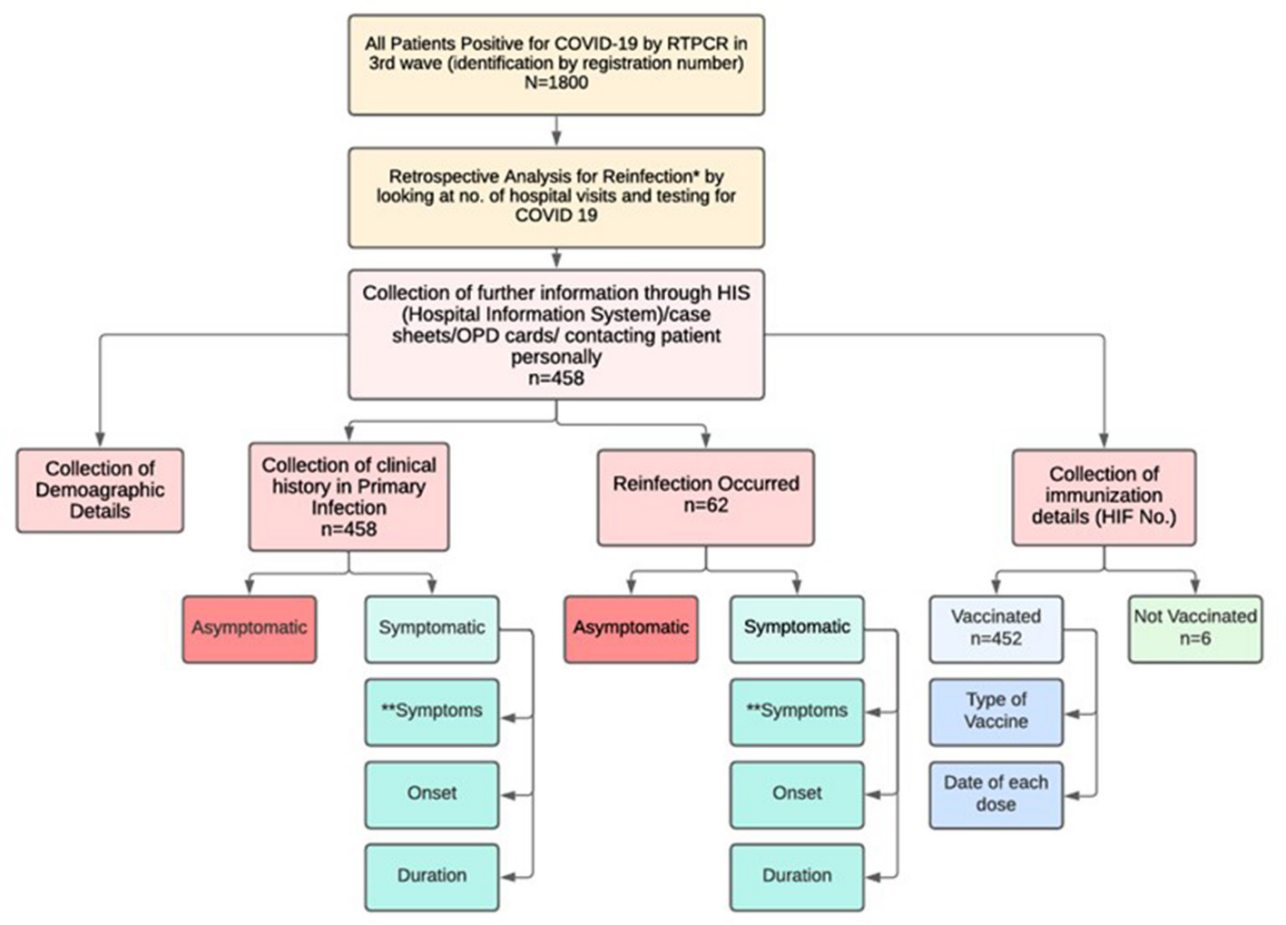
Data collection protocol. *Clinical recurrence of symptoms compatible with COVID-19, accompanied by positive RT PCR test (Ct < 35) or antigen test more than 90 days after the onset of the primary infection, supported by close-contact exposure or outbreak settings, and no evidence of another cause of infection. **Cough, dyspnea, chest pain, fever, headache, fatigue, myalgia, etc.

All patients tested positive for COVID-19 by RT-PCR or Antigen detection test during the third wave in January 2022. Patients who tested positive but were unwilling to share information or were not reachable were excluded from the study. Approximately 1,800 patients tested positive in January 2022 at the study center. Among the 1,800 patients, information about primary infection, reinfection, and vaccination status was obtained by referring to case records for inpatients. Patients who tested positive on an outpatient basis were called in person or sent a Google Form to gather information. The data collection protocol is illustrated in Figure 1.

Among the 1,800 patients who were initially eligible for follow-up, complete details could be obtained from only 458 individuals (25.4%). A large percentage of patients could not be contacted for multiple reasons, including registered phone numbers that were unreachable or switched off. Additionally, a considerable percentage of patients were unwilling to participate or declined to share personal or clinical information when contacted. As a result, only those patients for whom reliable data could be collected were included in the study.

Additionally, from this subgroup, patients who fulfilled the criteria for COVID-19 reinfection and visited the study setting were included, along with those who had experienced only a single episode of infection. Reinfection was defined as the clinical recurrence of symptoms compatible with COVID-19, accompanied by a positive RT-PCR test (Ct < 35) or antigen test more than 90 days after the onset of the primary infection, supported by documented close-contact exposure or occurrence in an outbreak setting, and with no alternative cause for infection.

The patients who visited our tertiary care hospital were given a unique registration number with which we could identify the number of times the patient had visited the hospital and the number of times the patient had undergone COVID-19 testing at our center. We also identified patients who had undergone reinfection by identifying them personally. Additional details were collected from the patients, entered into an Excel sheet, and used for analysis. Among the 1,800 patients who tested positive for COVID-19, detailed information could be collected for 458 (25.4%) patients, including both outpatients and inpatients.

The demographic details, clinical history and COVID-19 vaccination details were obtained through our hospital information system (HIS), by referring to case sheets or OPD cards of the patient, or by contacting them through their contact numbers mentioned in the HIS (Hospital information system) to obtain detailed information using a semi-structured questionnaire in the form of a proforma. The clinical details collected in symptomatic patients include clinical symptoms and signs, along with the duration of illness in both primary and reinfection. The vaccination details collected included the type of vaccine taken, date of administration, and whether they were not vaccinated as per the protocol.

### 2.1 Statistical analysis

Statistical Analysis was performed using SPSS software version 25. The demographic characteristics (age, sex, occupation, and comorbid conditions) of the patients were represented using arithmetic means, standard deviations, and percentages. The chi-squared test was used to determine the association between symptoms and infection status. Statistically significant was set at *p* < 0.05.

## 3 Results

Out of the 1,800 patients who were diagnosed with COVID-19 at the study center, we were able to retrospectively collect data from 458 individuals. Of these, 236 (51.5%) were males and 222(48.5%) were females. The average age of the participants was 39.1 ± 15.4 years, with a minimum of 2 years and a maximum of 91 years. We found that 36.68% of our study participants belonged to the age group range of 21–30 years. Of the 458 study participants, 62 (13.54%) with primary infection had re-infection, as confirmed by laboratory tests at the study center. The reinfection rate in the study area was 13.54% (Table 1).

**Table 1:** COVID-19 reinfection among study participants (*n* = 458) vaccination status, and vaccine types.

Characteristic	Infected once *n* (%)	Reinfected *n* (%)	Total *n* (%)
15.9-7.8,-1.4498.8ptOverall	396 (86.46)	62 (13.54)	458 (100)
Vaccination status			
Vaccinated (1 or 2 doses)	391 (98.74)	61 (98.39)	452 (98.69)
15.9-7.8,-1.4498.8ptNot vaccinated	5 (1.26)	1 (1.61)	6 (1.31)
Vaccine type			
Covishield	326 (82.32)	52 (83.87)	378 (82.52)
Covaxin	62 (15.66)	9 (14.52)	71 (15.50)
Covovax	2 (0.51)	0 (0)	2 (0.44)
Sputnik	1 (0.25)	0 (0)	1 (0.22)
Not vaccinated	5 (1.26)	1 (1.61)	6 (1.31)

Percentages for “Infected Once” and “Reinfected” columns are calculated within each group (*n* = 396 and *n* = 62, respectively). Total column percentages are calculated from the overall sample (*n* = 458).

Of the 458 COVID-19 patients, 452 were vaccinated (98.7%, 452/458) and six (1.31%, 6/458) were not vaccinated. Among the 62 reinfected COVID-19 patients, 61 (98.3%) were vaccinated and only 01 (1.7%) patient was not vaccinated. Among those who had primary infections, 391 (98.7%) were vaccinated, and five (1.3%) were not vaccinated. Among the study participants, 13.4% had re-infection (61/452), and 20% of those not vaccinated had re-infection (1/5), as shown in Table 1.

The study found that the most common vaccine taken by patients was COVISHIELD, a recombinant vaccine, covering 82.53%, that is, 378 of our study participants, followed by the vaccine COVAXIN, which is an inactivated vaccine covering 15.5%, that is, 71 of our study participants. Other vaccines included Covovax and Sputnik (Table 1).

The average duration between vaccination and infection among the study participants was 6.7 months. This was derived from 296 patients getting infected (primary) following both doses of vaccine, but complete vaccination details were not available for 66, so the average was taken for 230 patients, with the maximum duration being 12 months and the minimum being 1 month, as depicted in Table 2. The majority of infections occurred in the 4–8-month period post-vaccination (*n* = 90, 39.1%), followed by the 8–12- month period (*n* = 85, 37.0%), and the 1–4-month period (*n* = 55, 23.9%). This distribution pattern was consistent across vaccine types, particularly for Covishield recipients who comprised 84.8% of this cohort.

**Table 2:** Distribution of time from completion of vaccination to primary COVID-19 infection by vaccine type (*n* = 230).

Duration since Vaccination	Covishield	Covaxin	Covovax	Sputnik	Total
1–4 months	49	4	1	1	55 (23.9%)
4–8 months	74	15	1	0	90 (39.1%)
8–12 months	72	13	0	0	85 (37.0%)
Total	195	32	2	1	230^*^

^*^represents *n* = 230. Of the 391 vaccinated participants who experienced primary infection [Table 1], 95 were excluded as they had received only a single vaccine dose prior to infection. Among the remaining 296 fully vaccinated individuals, complete data on timing between vaccination completion and primary infection could not be established for 66 participants, yielding a final analytic sample of *n* = 230.

**Figure 2:**
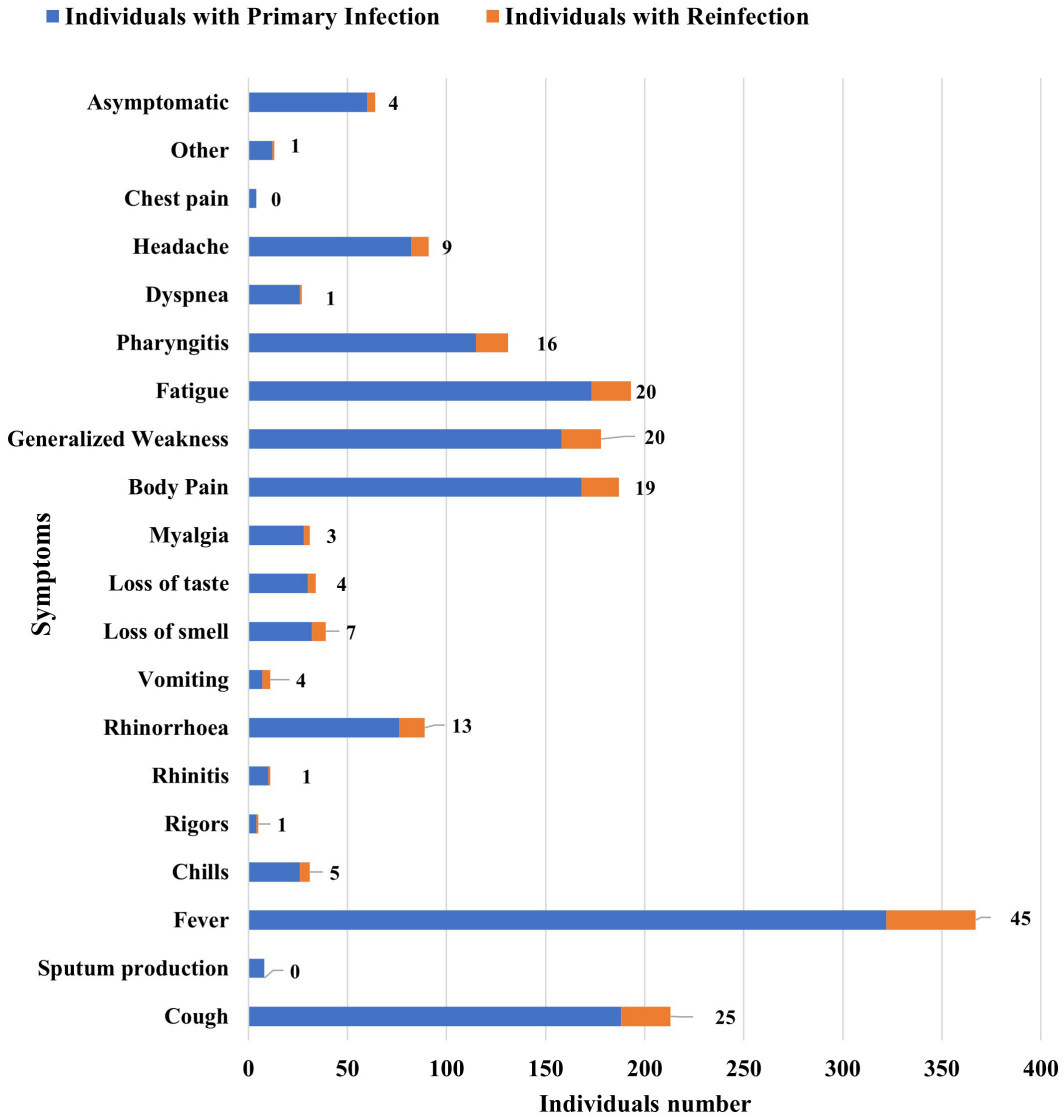
Distribution of patients based on various symptoms in primary infection and reinfection.

The patients who tested positive in January 2022 at the study center were largely outpatients (441 out of 458), accounting for 96%. Seventeen patients were admitted to hospitals in various COVID wards and ICUs, accounting for 4%. Of the 458 people infected with COVID-19 for the first time, 60 (13%) were asymptomatic and 398 (87%) were symptomatic. The most frequently observed symptom was fever, observed in 322 patients (70.3%). The next most common symptom was a cough, followed by body pain and generalized weakness. Chest pain and rigors were seen the least, with only four people presenting with these symptoms each (0.87%), as depicted in Table 3 and Figure 2.

**Table 3:** Distribution of patients based on various symptoms in primary infection and reinfection cases.

Symptoms	Individuals with primary infection		Individuals with reinfection	
	Numbers	Percentage	Numbers	Percentage
Cough	188	41.04	25	40.32
Sputum production	8	1.74	0	0
Fever	322	70.30	45	72.58
Chills	26	5.67	5	8.06
Rigors	4	0.87	1	1.61
Rhinitis	10	2.18	1	1.61
Rhinorrhoea	76	16.59	13	20.96
Vomiting	7	1.52	4	6.45
Loss of smell	32	6.98	7	11.29
Loss of taste	30	6.55	4	6.45
Myalgia	28	6.11	3	4.83
Body pain	168	36.68	19	30.64
Generalized weakness	158	34.49	20	31.25
Fatigue	173	37.77	20	31.25
Pharyngitis	115	25.10	16	25.80
Dyspnea	26	5.67	1	1.61
Headache	82	17.90	9	14.51
Chest pain	4	0.87	0	0
Other	12	2.62	1	1.61
Asymptomatic	60	13.10	4	6.45

Chi-square analysis was done to determine the association of symptoms of COVID-19 with the status of infection. A statistically significant association was observed between vomiting and infection status (*p* = 0.011). Vomiting was more frequently reported during reinfection (6.5%) compared to primary infection (1.6%). The other symptoms did not show a statistically significant association (Table 4).

**Table 4:** Distribution of study participants based on the symptoms during the primary and re-infection, and the association of symptoms with the status of infection.

Symptoms	Status of infection (*N* = 520)		Chi-square value/Fisher's exact value	*p-value*
	Primary infection (*n* = 458)	Re-infection (*n* = 62)		
Asymptomatic	60 (13.11)	4 (6.45)	2.2	0.13
Symptomatic	398 (86.89)	58 (93.55)		
Cough			0.012	0.91
No	270 (58.96)	37 (59.67)		
Yes	188 (41.04)	25 (40.32)		
Sputum production			1.1	0.29
No	450 (98.26)	62 (100)		
Yes	08 (1.74)	0		
Fever			0.13	0.71
No	136 (29.7)	17 (27.41)		
Yes	322 (70.30)	45 (72.59)		
Chills			0.55	0.45
No	432 (94.33)	57 (91.93)		
Yes	26 (5.67)	5 (8.07)		
Rigor			0.31	0.57
No	454 (99.13)	61 (98.38)		
Yes	04 (0.87)	01 (1.62)		
Rhinorrhea			0.73	0.39
No	382 (83.41)	49 (79.03)		
Yes	76 (16.59)	13 (20.97)		
Rhinitis			0.086	0.77
No	448 (97.82)	61 (98.38)		
Yes	10 (2.18)	01 (1.62)		
Vomiting			6.3	0.011
No	451 (98.4)	58 (93.5)		
Yes	07 (1.6)	04 (6.5)		
Loss of smell			1.4	0.22
No	426 (93.02)	55 (88.70)		
Yes	32 (6.98)	07 (11.3)		
Loss of taste			0.001	0.97
No	428 (93.45)	58 (93.54)		
Yes	30 (6.55)	04 (6.46)		
Myalgia			0.15	0.6
No	430 (93.89)	59 (95.16)		
Yes	28 (6.11)	03 (4.84)		
Body pain			0.86	0.35
No	290 (93.32)	43 (69.35)		
Yes	168 (36.68)	19 (30.65)		
Generalized weakness			0.12	0.7
No	300 (65.51)	42 (67.74)		
Yes	158 (34.49)	20 (32.26)		
Fatigue			0.7	0.39
No	285 (62.23)	42 (67.74)		
Yes	173 (37.77)	20 (32.26)		
Pharyngitis			0.014	0.9
No	343 (74.9)	46 (74.19)		
Yes	115 (25.10)	16 (25.81)		
Dyspnea			1.8	0.17
No	432 (94.33)	61 (98.38)		
Yes	26 (5.67)	1 (1.62)		
Headache			0.43	0.51
No	376 (82.1)	53 (85.48)		
Yes	82 (17.90)	09 (14.52)		
Chest pain			0.54	1^*^
No	454 (99.13)	62 (100)		
Yes	04 (0.87)	0		
Other symptoms			0.22	0.63
No	446 (97.38)	61 (98.38)		
Yes	12 (2.62)	1 (1.62)		

Legend: ^*^Fisher's exact test numbers in parentheses represent percentages column-wise.

## 4 Discussion

Severe acute respiratory syndrome coronavirus 2 (SARS-CoV-2), the 17 most common human coronavirus, is the causative agent of COVID-19. Patients with SARS-CoV-2 infection may present with a range of symptoms from mild to severe. The major challenge in controlling the current pandemic is the presence of a large population of asymptomatic carriers. Early diagnosis, complete vaccination, and optimum infection control measures are the major ways to control the pandemic. In our study, we found that COVID-19 affected adults in the age group of 21–30 years, followed by the age group of 30–40 years, compared to other age groups. This may be due to the increased chances of exposure of the working population. A study in the US has reported reinfections to be more common in the age group of 18–49 years compared to in those aged more than 50 years (4).

In the current study, it was observed that males and females were infected almost equally with males having a slightly greater prevalence in our study (52%) which is similar to a study by Yadav et al., on Association of Gender, Age, and Comorbidities with COVID-19 infection in India where they reported a higher burden of COVID-19 among male patients at 50.3% (5). According to an article by Ajay Pradhan on “Sex differences in severity and mortality from COVID-19: are males more vulnerable?” showed that the severity and mortality of COVID-19 were higher in males than in females. Some studies have shown that this may be due to delayed viral clearance in males due to the high expression of ACE2 in the testis compared to the ovaries. Another influencing factor may be due to immunosuppression by testosterone in males and the immunoenhancing effect of estrogen in females. Other contributing factors may be the presence of other underlying diseases that occur more commonly in males, such as heart or liver disease, diabetes, and cancer (6).

This study reports the rate of reinfection to be 13.54%, with 62 individuals reinfected out of 458. A study conducted in Delhi showed a reinfection rate of 28.4% among healthcare workers (1,007/3,545) (7). The reinfection rate varies with geographical location and depends on various factors, such as vaccination and protection following primary infection. The present study also showed that most of the reinfected patients had mild illness compared to primary infection, with only one person suffering from moderate illness during reinfection. A similar Retrospective Cohort study was conducted by Megan M. Sheehan, Anita J. Reddy, and Michael B. Rothberg on 150,325 patients (8). They found that prior infection was highly protective against reinfection and symptomatic diseases. This indicates that primary infection or vaccination offers good protection.

We further found that COVISHIELD, which is a recombinant vaccine, was the most widely used vaccine (82.53%), followed by COVAXIN, which is an inactivated vaccine (15.50%). This is similar to a study by Bhardwaj et al. (9), which reported similar vaccination patterns in India with 80.9% of their vaccinated individuals vaccinated with Covishield and 19.1% with Covaxin. In contrast, a study conducted in Northern India among healthcare workers revealed that 88% of them had received Covaxin and 11% were vaccinated with Covishield (7). Irrespective of the type of vaccine received, vaccinated individuals are protected against COVID-19. If they develop an infection, they tend to have a milder illness and the risk of mortality is significantly reduced. The number of unvaccinated individuals in the study was very low (1.31%), showing good coverage and vaccine distribution. In this study, 20% (1 in 6) non vaccinated participants compared to 13.5% (61 in 452) vaccinated participants had re-infection, indicating that vaccination protects against reinfection and severe illness as shown in a study by Arora et al. (10), which demonstrated reduced breakthrough infections and severity among vaccinated individuals in Delhi.

We found that the average duration of protection after the second dose of the vaccine until the primary infection was 6.7 months. The higher proportion of infections in the 4–12-month window (76.1%) compared to 1–4 months (23.9%) may suggest waning vaccine-induced immunity over time, supporting the rationale for booster dose administration. However, these findings should be interpreted cautiously as they may also reflect temporal variations in community transmission rates or the emergence of immune-evasive variants during the study period.

A study conducted in the United Kingdom revealed that vaccine effectiveness against symptomatic disease due to the delta variant peaked in the early weeks following the receipt of the second dose and then decreased by 20 weeks (11).

Our study indicated that the most common symptom seen in both primary infection and reinfection was fever followed by cough, similar to what was seen in a similar study conducted elsewhere, and found that 98% of the general population had fever, 76% had cough, 55% had dyspnea, and up to 44% had myalgia or fatigue (12). In our study, we found fever in 70.3% of individuals and cough in 41.04% of patients with the primary infection. Weakness and body pain were observed in 34.49% and 36.68% of the patients, respectively. Shortness of breath was not observed as much as it was only present in 5.67% of patients. In the reinfection group, fever was present in 72.58%, cough in 40.32%, and weakness and body pain were both just above 30%. The study by Bhardwaj et al. (9) similarly found fever to be the most prevalent symptom over multiple waves. They also found the percentage of fever, shortness of breath, headache, abdominal pain, cold/running nose, and fatigue to be highest in Wave-I as compared to Wave-II. Statistical analysis revealed a significant association between vomiting symptoms and infection status (*p* < 0.01). Previous studies have revealed that nausea and vomiting are common symptoms observed in both adults and children, and their presence can be the initial symptom of SARS-CoV-2 infection (13). Gastrointestinal symptoms in COVID-19 may be rare; however, they are significantly associated with COVID-19.

In the third wave of COVID-19, most of the patients visited the hospital on an outpatient basis (OP) in contrast to the second wave, which was caused by the Delta variant of SARS COv-2, during which a high rate of admission to various wards and ICUs of the hospitals was observed. This difference may be due to the infection caused by a less virulent Omicron strain during the third wave. A large population was asymptomatic or had mild illnesses for which they were treated on an outpatient basis in the third-wave findings, which is in concordance with other studies (14). Similarly, the study by Bhardwaj et al. compared symptoms and severity across multiple COVID-19 waves, where they found the proportion of symptomatic infection declined from Wave-I to Wave-III (9).

The number of reinfected cases was 62 of 458. Thus, the actual rate of reinfection may have been higher. However, due to the lack of RT-PCR testing post-first infection, as people may be under the misassumption that primary infection confers complete immunity, they may not have been detected and diagnosed. The presence of asymptomatic or mild illness during the third wave could be another reason why people were not being tested. The average duration of protection provided by infection for those who were reinfected could not be calculated, as data for the date of reinfection could not be collected for all. The sequelae of SARS-CoV-2 infection can sometimes be mistaken for reinfection, including persistent symptoms and/or complications observed in patients beyond 4 weeks of symptoms.

### 4.1 Limitations

- This study has a few limitations. First, the number of confirmed reinfection cases was relatively small, which limited the statistical power to perform robust multivariable analyses to control for confounders such as age, comorbidities, and time since vaccination.
- Some clinical and vaccination details were incomplete due to the retrospective design and reliance on hospital records and patient recall.
- Reinfection was defined based on clinical and testing criteria without genomic sequencing to confirm strain differences. Finally, as a short report from a single center, the findings may have limited generalizability.

## 5 Conclusion

COVID-19 patients, although presenting with characteristic respiratory signs and symptoms, present with gastrointestinal symptoms or other system involvement have become a challenge for accurate and early clinical diagnosis. In our study, the symptoms of COVID-19 infection during primary and re-infection did not differ to a great extent. In addition, vaccination has proven to be an effective method to prevent transmission, suggesting that repeated vaccination with booster doses that are modified based on circulating variants is essential for curbing the pandemic along with new surges, preventing further reinfections with different strains, and preventing waning of vaccine efficacy.

## Data Availability

The original contributions presented in the study are included in the article/supplementary material, further inquiries can be directed to the corresponding author.

## References

[1] Silva DL Lima CM Magalhães VCR Baltazar LM Peres NTA Caligiorne RB . Fungal and bacterial coinfections increase mortality of severely ill COVID-19 patients. J Hosp Infect. (2021) 113:145–54. doi: 10.1016/j.jhin.2021.04.001 33852950 PMC8056850

[2] Yahav D Yelin D Eckerle I Eberhardt CS Wang J Cao B . Definitions for coronavirus disease 2019 reinfection, relapse and PCR re-positivity. Clin Microbiol Infect. (2021) 27:315–8. doi: 10.1016/j.cmi.2020.11.028 33285276 PMC7718119

[3] Wang J Kaperak C Sato T Sakuraba A. COVID-19 reinfection: a rapid systematic review of case reports and case series. J Investig Med. (2021) 69:1253–5. doi: 10.1136/jim-2021-001853 34006572

[4] Ma KC Dorabawila V León TM Henry H Johnson AG Rosenberg E . Trends in laboratory-confirmed SARS-CoV-2 reinfections and associated hospitalizations and deaths among adults Aged≥ 18 Years −18 US Jurisdictions, September 2021–December 2022. MMWR Morb Mortal Weekly Rep. (2023) 72:683–9. doi: 10.15585/mmwr.mm7225a3 PMC10328471 37347715

[5] Yadav SK Bhardwaj P Gupta P Saluja D Jetly S Taneja J. Association of gender, age, and comorbidities with COVID-19 infection in India. J Integr Sci Technol. (2022) 10:61–6. doi: 10.62110/sciencein.jist.2022.v10.61

[6] Pradhan A Olsson PE. Sex differences in severity and mortality from COVID-19: are males more vulnerable? Biol Sex Diff . (2020) 11:1–1. doi: 10.1186/s13293-020-00330-7 32948238 PMC7498997

[7] Malhotra S Mani K Lodha R Bakhshi S Mathur VP Gupta P . COVID-19 infection, and reinfection, and vaccine effectiveness against symptomatic infection among health care workers in the setting of omicron variant transmission in New Delhi, India. Lancet Region Health Southeast Asia. (2022) 3:100023. doi: 10.1016/j.lansea.2022.100023 35769163 PMC9167830

[8] Sheehan MM Reddy AJ Rothberg MB. Reinfection rates among patients who previously tested positive for coronavirus disease 2019: a retrospective cohort study. Clin Infect Dis. (2021) 73:1882–6. doi: 10.1093/cid/ciab234 33718968 PMC7989568

[9] Bhardwaj P Jetly S Yadav S Kaushik R Naidu K Saluja D . Comparative analyses of symptoms, severity, and breakthrough infections in vaccinated and unvaccinated individuals during three waves of COVID-19 in India. Acta Virol. (2024) 68:13536. doi: 10.3389/av.2024.13536

[10] Arora G Taneja J Bhardwaj P Goyal S Naidu K Yadav SK . Adverse events and breakthrough infections associated with COVID-19 vaccination in the Indian population. J Med Virol. (2022) 94:3147–54. doi: 10.1002/jmv.27708 35261064 PMC9088477

[11] Andrews N Tessier E Stowe J Gower C Kirsebom F Simmons R . Duration of protection against mild and severe disease by Covid-19 vaccines. New Engl J Med. (2022) 386:340–50. doi: 10.1056/NEJMoa2115481 35021002 PMC8781262

[12] Shahid Z Kalayanamitra R McClafferty B Kepko D Ramgobin D Patel R . COVID-19 and older adults: what we know. J Am Geriatr Soc. (2020) 68:926–9. doi: 10.1111/jgs.16472 32255507 PMC7262251

[13] Zhang T Liu D Tian D Xia L. The roles of nausea and vomiting in COVID-19: did we miss something? J Microbiol Immunol Infect. (2021) 54:541–6. doi: 10.1016/j.jmii.2020.10.005 34435559 PMC7568482

[14] Ranjan R. Omicron impact in India: an early analysis of the ongoing COVID-19 third wave. MedRxiv [Preprint]. (2022). doi: 10.1101/2022.01.09.22268969

